# Third-Generation Sirolimus‐Eluting Bioresorbable Tyrocore Scaffold Implantation in Patients with ST‐Segment Elevation Myocardial Infarction: Baseline and 6-Month OCT and Clinical Outcomes—a FANTOM STEMI Pilot Study

**DOI:** 10.1007/s10557-023-07429-0

**Published:** 2023-01-14

**Authors:** Lukasz Koltowski, Mariusz Tomaniak, Dorota Ochijewicz, Grzegorz Opolski, Janusz Kochman

**Affiliations:** 1st Department of Cardiology, Medical University of Warsaw, Banacha 1a, 02-097 Warsaw, Poland

**Keywords:** BVS, STEMI, OCT, Long-term

## Abstract

**Purpose:**

The aim of this study was to evaluate the safety and efficacy of the Fantom BRS 6 months after implantation using the optical coherence tomography (OCT) imaging.

**Methods:**

Twenty STEMI patients treated with a sirolimus‐eluting Fantom BRS were enrolled into a prospective, single-arm, serial observational study. The scaffold sizing, positioning and optimisation were guided by OCT imaging. The primary endpoint was device‐orientated composite endpoints (DOCE), comprised of cardiac death, target-vessel-related myocardial infarction and target lesion failure. To evaluate the device performance at the scaffold level, we performed a quantitative coronary angiography (QCA) and OCT imaging at 6 months.

**Results:**

The primary endpoint did not occur in any patient within the 6-month follow‐up. There were no major adverse cardiac events (MACEs) or DOCEs, no cases of scaffold thrombosis, target lesion revascularization and no deaths. In QCA, we observed a decrease in the minimum and mean lumen diameter in the in-scaffold region and in the proximal and distal peri-scaffold region. Similarly, the minimum lumen area and reference vessel diameter had decreased in both QCA and OCT. The OCT imaging showed improvement in the expansion index and malposition rate.

**Conclusion:**

A serial 6-month OCT imaging after implantation of a third-generation Tyrocore-based bioresorbable coronary scaffold indicated good coverage of the struts with excellent healing of the scaffold, low neointima growth and no signs of neoatherosclerosis.

## Introduction 

Upon its introduction, the bioresorbable vascular scaffold (BVS) (Absorb™; Abbott Vascular, Santa Clara, CA, USA) was declared a transformative technology designed to restore coronary structure and functionality and to enhance the long-term outcomes of metallic stents by overcoming the dangers of stent thrombosis, stent fracture and neoatherosclerosis. These hopes were not confirmed by the early and very late results of randomised controlled trials that compared the BVS with the XIENCE everolimus-eluting stent (Abbott Vascular), which demonstrated increased rates of scaffold thrombosis and target lesion failure (TLF) after 1, 2, 4 and 5 years [1–4]. These safety concerns led to a cessation of the commercialisation of the product and to the European Society of Cardiology guidelines downgrading the recommendation for the use of BVSs to class III [5]. Still, insights from other clinical programmes evaluating next-generation coronary scaffolds suggest that improvement of the technology, especially in regard to the mechanical and biocompatibility properties of the polymer, is needed to justify its clinical use [6–8]. The Fantom (REVA Medical, USA) is a third-generation bioresorbable scaffold (BRS) made with Tyrocore, a new polymer composed mainly of an iodinated short-chain polycarbonate copolymer of tyrosine analogues and characterised by less release of lactic acid, less irritation, much-diminished tissue calcium formation and improved endothelisation in comparison to Absorb [9]. Long-term follow-up of Fantom BRS in chronic coronary syndrome showed sustained safety and efficacy (TLF = 5.4%; major adverse cardiac events [MACE] = 5.8%) after 48 months) that is comparable to contemporary drug-eluting stents (DES) [10].

ST-segment elevation myocardial infarction (STEMI) has been identified as one of the more favourable scenarios for the use of BVS technology for primary percutaneous coronary intervention (pPCI), since (1) the patients are usually younger at admission, with a high likelihood of repeat intervention over the lifespan, and thus may have greater benefit from cage-free coronary vessels, (2) their plaques are ruptured, soft and thrombotic, and (3) the strong mechanical support of the vessel wall is less of importance as the fibro-calcific morphology is rare [11].

To date, no clinical studies have assessed arterial healing of third-generation Fantom Encore BRS implantation in the thrombogenic milieu of a STEMI setting. Therefore, the purpose of the Fantom STEMI pilot project was to obtain a serial prospective follow-up and to evaluate the healing process over 36 months after pPCI. Herein, we present the results of the early phase after a 6-month clinical, angiographic and intravascular imaging follow-up was conducted.

## Methods

### Device, Patients, and Study Design

This is a single-centre, investigator-initiated prospective registry of STEMI patients (clinicaltrial.org, NCT03785431) treated with the Fantom device. The Fantom is the third-generation balloon-expandable, CE-marked BRS. It is characterised by a thin strut profile (95–115 μm) and is made of Tyrocore with enhanced radiopacity. The details of the device have been described previously [12].

At baseline, 20 patients underwent optical coherence tomography (OCT)-guided pPCI with Fantom Encore implantation. The inclusion and exclusion criteria as well as the procedural protocol have been reported elsewhere [12]. In brief, adults with typical chest pain onset of less than 12 h and electrocardiographic confirmation of ST-segment elevation were recruited. The angiographic inclusion criteria included a de novo lesion in a native coronary artery and visually estimated stenosis of at least 50% with a reference vessel diameter (RVD) of between 2.5 and 3.5 mm and a lesion length of up to 20 mm. The exclusion criteria were cardiogenic shock, pulmonary oedema, known hypersensitivity to or contraindications for antiplatelet/antithrombotic medications or contrast agents and specific lesion locations or characteristics (non-native vessel, left main coronary artery, true bifurcation and significant calcifications). A 12-month dual antiplatelet therapy with aspirin and ticagrelor was recommended.

**Fig. 1:**
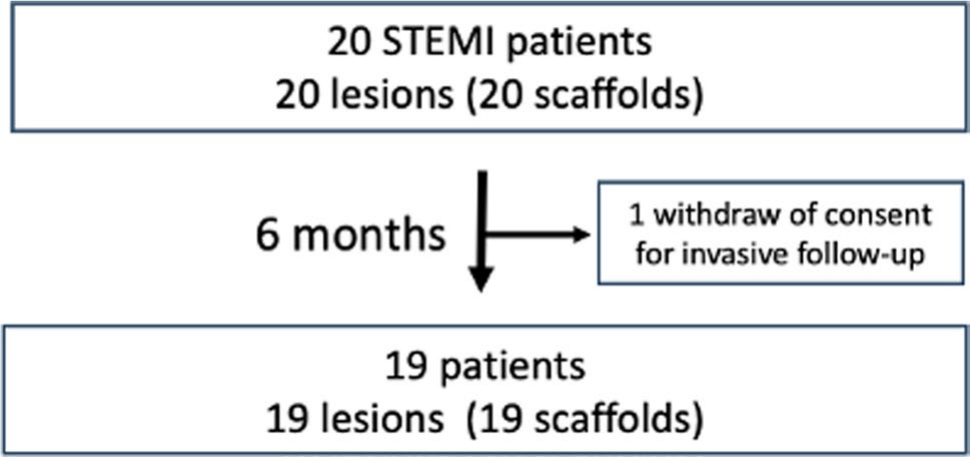
Study flow chart. STEMI, ST segment elevation myocardial infarction

At 6 months, the patients were scheduled for hospital admission for clinical and invasive assessment with coronary angiography and intravascular imaging using OCT. The study flow chart is presented in Fig. 1. The local ethics committee approved the protocol following the Declaration of Helsinki.

### Study Endpoints and Definitions

The study endpoints have been described previously [12]. In brief, the primary device-orientated composite endpoints (DOCE) consisted of cardiac death, target-vessel MI or ischemia-driven TLR at 180 days. MACEs included nonfatal MI, TLR, target-vessel revascularization (TVR) and cardiovascular death. Patient-orientated composite endpoints (POCE) consisted of all-cause mortality, any myocardial infarction and repeat revascularisation [13]. Additionally, an angiographic and OCT analysis were performed.

### Baseline Procedure and Follow-Up Intravascular Imaging

The pPCI was performed according to the previously published study protocol [12]. In brief, the patients provided informed consent in the CathLab before the procedure. Both radial and femoral access were allowed. The angiographic eligibility criteria were checked in the 2 near orthogonal projections of the target segment. At the operator’s discretion, manual aspiration thrombectomy or an undersized balloon to restore the blood flow (at least TIMI 2) were allowed. An OCT pullback was performed for sizing and lesion morphology assessment, followed by predilatation with a 1:1 noncompliant or semicompliant balloon. Post-dilatation was mandatory to achieve optimal scaffold expansion in OCT. At 6 months, an in-hospital invasive follow-up with intravascular OCT and angiography analysis was carried out in all patients.

### Angiographic and OCT Imaging Analysis

An off-line qualitative and quantitative coronary angiography (QCA) and OCT were analysed by an independent core laboratory (Krakow Cardiovascular Research Institute, Krakow, Poland) using previously reported methodology [12]. In brief, the following QCA parameters were assessed in the scaffolded segment: RVD, minimal lumen diameter (MLD), diameter stenosis (%DS), minimum lumen area and late lumen loss (LLL).

The OCT imaging was performed with a Dragonfly™ OPTIS™ catheter (Abbott Vascular, Santa Clara, CA, USA) according to standard procedures and was analysed at an independent core laboratory (Krakow Cardiovascular Research Institute, Krakow) by analysts blinded to the angiographic data and clinical characteristics using proprietary LightLab off-line analytical software. The analysis was performed in the in-scaffold segments (IS) and out-scaffold segments (OS) (5 mm adjacent to the proximal and distal segments from the radiopaque platinum markers) [14]. The qualitative and quantitative OCT analyses are described in the Supplementary Appendix.

## Statistical Analysis

The primary endpoint and all imaging findings (OCT and angiography) were analysed based on the as-treated population. The data distribution was assessed by Kolmogorov–Smirnov analysis. The continuous variables are presented as mean ± standard deviation (SD) for those with normal distributions or as median and interquartile range (IQR) for those with non-normal distribution. The categorical variables are presented as counts and percentages. The Wilcoxon signed-rank test was used for 2 related sample comparisons; the Pearson correlation coefficient was used for continuous variables. Mixed models were used to consider the clustered nature of OCT data regarding struts, malposition, segments and edges. Statistical analysis was performed using JMP software, version 9.0.0 (SAS Institute, Cary, NC, USA).

## Results

Overall, 20 patients were enrolled in the study, and 19 patients underwent clinical and invasive follow-up at 6 months. One patient, who did not report any MACEs and felt well, withdrew their informed consent for invasive imaging (Fig. 1). The demographic and clinical characteristics at baseline are shown in Table 1. The mean age was 61.26 ± 9.48 years; there were 9 women (47.4%).

**Table 1:** Baseline characteristics (*n* = 19)

Age (years)	61.26 ± 9.48
Gender, male	10 (52.6%)
BMI (m^2^/kg)	28.78 ± 3.95
Vital signs on admission	
HR (bpm)	79.58 ± 17.87
SBP (mm Hg)	141.37 ± 28.01
DBP (mm Hg)	77.89 ± 13.47
Risk factors	
Family history of CAD	5 (26.3%)
Hypertension	14 (73.7%)
Hyperlipidaemia	4 (21.1%)
Impaired renal function	1 (5.3%)
Peripheral vascular disease	0 (0%)
Prior PCI	0 (0%)
Prior CABG surgery	0 (0%)
Prior MI	0 (0%)
Congestive heart failure	1 (5.3%)
History of smoking	13 (68.4%)
Current smoker	7 (36.8%)
Prior smoker	6 (31.6%)
Diabetes	4 (21.1%)
Gastrointestinal bleeding	0 (0%)
Stroke/TIA	1 (5.3%)
Region of ST-segment elevation	
Inferior wall	8 (42.1%)
Anterior wall	8 (42.1%)
Lateral wall	3 (15.8%)
Laboratory analysis, at admission	
RBC	4.70 ± 0.57
WBC	11.46 ± 2.99
HCT	42.10 ± 4.11
HGB	14.48 ± 1.59
Platelets	249.05 ± 55.80
Creatinine	0.95 [0.76, 1.04]
Urea	40.47 ± 17.32
ALT	37.06 ± 17.29
AST	61.29 ± 98.31
LDL	109.38 ± 23.38
HDL	46.79 ± 13.02
Triglycerides	159.64 ± 68.35
Total cholesterol	175.80 ± 38.68
Fasting glucose level	127.69 [99.5, 133.0]
Thyroid stimulation hormone	2.39 ± 2.52
Left ventricular ejection fraction by echocardiography, %	48.11 ± 8.26
Medications on admission	
Beta blockers	6 (31.6%)
Anti-arrhythmic	0 (0%)
Aldosterone antagonists	0 (0%)
Nitrates	0 (0%)
Diuretics	4 (21.1%)
ACE inhibitors	7 (36.8%)
Antiplatelet agents	0 (0%)
Anticoagulants/thrombolytics	0 (0%)

Data are presented as mean ± standard deviation, number (%) or median [first quartile, third quartile]

*BMI*, body mass index; *CABG*, coronary artery bypass grafting; *CAD*, coronary artery disease; *DSP*, diastolic blood pressure; *HR*, heart rate; *MI*, myocardial infarction; *PCI*, percutaneous coronary intervention; *SBP*, systolic blood pressure

The details of the procedures are summarized in Table 2. The procedures were performed via radial (*n* = 17; 89.5%) and femoral (*n* = 2; 10.5%) access. A single scaffold was used in each patient. Manual aspiration thrombectomy was performed in 2 cases (10.5%). The mean stent diameter was 2.42 ± 0.56 mm, and the mean stent length was 14.42 ± 2.43 mm. The actual mean deployment pressure was 10.32 ± 2.78 atm. All patients received a dual antiplatelet therapy consisting of aspirin (100%); 13 patients received ticagrelor (68.4%), while the remaining 6 patients received a P2Y12 inhibitor, that is, clopidogrel (31.6%). An unfractionated heparin (UFH) was used in all cases.

**Table 2:** Details of the procedures (*n* = 19)

Antiplatelet therapy	
Aspirin	19 (100%)
Clopidogrel	6 (31.6%)
Ticagrelor	13 (68.4%)
Antithrombotic agent	
UFH	19 (100%)
Antithrombotic dose (units)	8158 ± 2192
Access site	
Right radial artery	17 (89.5%)
Right femoral artery	2 (10.5%)
Vessel	
RCA	7 (36.8%)
LAD	10 (52.6%)
LCx	2 (10.5%)
Sheath size	
6Fr	19 (100%)
Diameter stenosis (%)	95.42 ± 8.09
Reference vessel diameter (mm)	3.19 ± 0.36
Lesion length (mm)	16.06 ± 4.27
TIMI score, baseline	
0	10 (52.6%)
1	2 (10.5%)
2	6 (31.6%)
3	1 (5.3%)
Thrombectomy	2 (10.5%)
Pre-dilatation performed	19 (100%)
Predilatation balloon	
Diameter [mm]	2.42 ± 0.56
Length [mm]	14.42 ± 2.43
Pressure [25]	12.11 ± 2.54
Time (s)	14.47 ± 2.29
Diameter stenosis after pre-dilatation (%)	35.21 ± 36.89
TIMI Score after pre-dilatation	
0	0 (0%)
1	0 (0%)
2	4 (21.1%)
3	15 (78.9%)
Target corresponding inflation pressure [atm]	8.84 ± 2.24
Nominal diameter of scaffold [mm]	3.16 [3.0–3.5]
Nominal length of scaffold [mm]	21.16 [18.0–24.0]
Actual deployment pressure [atm]	10.32 ± 2.78
Residual diameter stenosis after scaffold deployment (%)	11.67% ± 4.85
Dissection noted after scaffold deployment	0 (0%)
Scaffold post-dilation	18 (94.7%)
Post-dilatation balloon	
Post-dilatation with NC balloon	17 (100%)
Balloon diameter [mm]	3.42 ± 0.40
Balloon length [mm]	11.44 ± 3.11
Pressure [atm]	17.67 ± 3.23
Time [s]	14.07 ± 2.25
Final diameter stenosis (%)	5.00 ± 2.78
Dissection noted after post-scaffold dilatation	0 (0%)
Edge dissection	0 (0%)
Final TIMI score	
0	0 (0%)
1	0 (0%)
2	1 (5.3%)
3	18 (94.7%)

Data are presented as mean ± standard deviation, number (%) or median [first quartile, third quartile]

*Cx*, circumflex artery; *Dg*, diagonal branch; *LAD*, left anterior descending artery; *Mg*, marginal branch; *NC*, non-compliant; *RCA*, right coronary artery; *TIMI*, thrombolysis in myocardial infarction; *UFH*, unfractioned heparin

Overall, at the 6-month follow-up, there were no events of the primary endpoint, defined as DOCE (cardiac death, target-vessel MI or TLR). No MACEs (MI, TLR, TVR or cardiac death) or cases of scaffold thrombosis were reported either. A POCE (death, MI or repeat PCI) was reported in 1 patient who underwent elective staged PCI of the left anterior descending artery with DES implantation. The details are presented in Table 3.

**Table 3:** Clinical outcomes at 180 days (*n* = 19)

DOCE (primary endpoint), *n* (%)	0 (0)
Cardiac death, *n* (%)	0 (0)
ID-TLR, *n* (%)	0 (0)
TV-MI, *n* (%)	0 (0)
Define/probable scaffold thrombosis, *n* (%)	0 (0)
MACE	0 (0)
POCE	1 (5.3%)

*DOCE*, device-orientated clinical endpoint; *ID-TLR*, ischemia-driven target lesion revascularisation; *MACE*, major adverse clinical event; *POCE*, patient-orientated clinical outcome; *TV-MI*, target vessel myocardial infarction

### Quantitative Coronary Angiography Analysis

The results from QCA analysis at baseline and at 180 days are shown in Table 4. We observed a statistically significant increase in %DS (5.28% ± 4.40% vs 9.39% ± 5.78%, *p* < 0.05) with a decrease in MLD (2.88 ± 0.35 mm vs 2.68 ± 0.38 mm, *p* < 0.01), minimum lumen area (6.58 ± 1.53 mm^2^ vs 5.74 ± 1.52 mm^2^, *p* < 0.01) and RVD (3.04 ± 0.36 mm vs 2.95 ± 0.36 mm, *p* < 0.05). The LLL at 6 months was 0.20 ± 0.25 mm.

**Table 4:** Paired quantitative coronary angiography analysis (scaffold level, *n* = 19)

Variable	Baseline (post-procedure)	6-month	*p*-value
Number of lesions	18 (90)	18 (90)	-
Lesion length [mm]	20.19 ± 3.40	20.49 ± 3.67	0.1613
In-scaffold segment			
Minimum lumen diameter [mm]	2.88 ± 0.35	2.68 ± 0.38	0.0035*
Mean lumen diameter [mm]	3.10 ± 0.32	2.91 ± 0.31	0.0017*
Diameter stenosis [%]	5.28 ± 4.40	9.39 ± 5.78	0.0153*
Acute gain [mm]	1.47 ± 0.51	-	-
Late lumen loss [mm]	-	0.20 ± 0.25	-
Minimum lumen area [mm^2^]	6.58 ± 1.53	5.74 ± 1.52	0.0043*
Peri-scaffold segment			
Reference vessel diameter [mm]	3.04 ± 0.36	2.95 ± 0.36	0.0427*
Proximal mean lumen diameter [mm]	3.19 ± 0.35	3.03 ± 0.33	0.0027*
Distal mean lumen diameter [mm]	2.84 ± 0.48	2.73 ± 0.44	0.0762

Data are presented as mean ± standard deviation, number (%) or median [first quartile, third quartile] unless otherwise indicated

^*^ *p*-value < 0.05

### Quantitative and Qualitative OCT Analysis

**Fig. 2:**
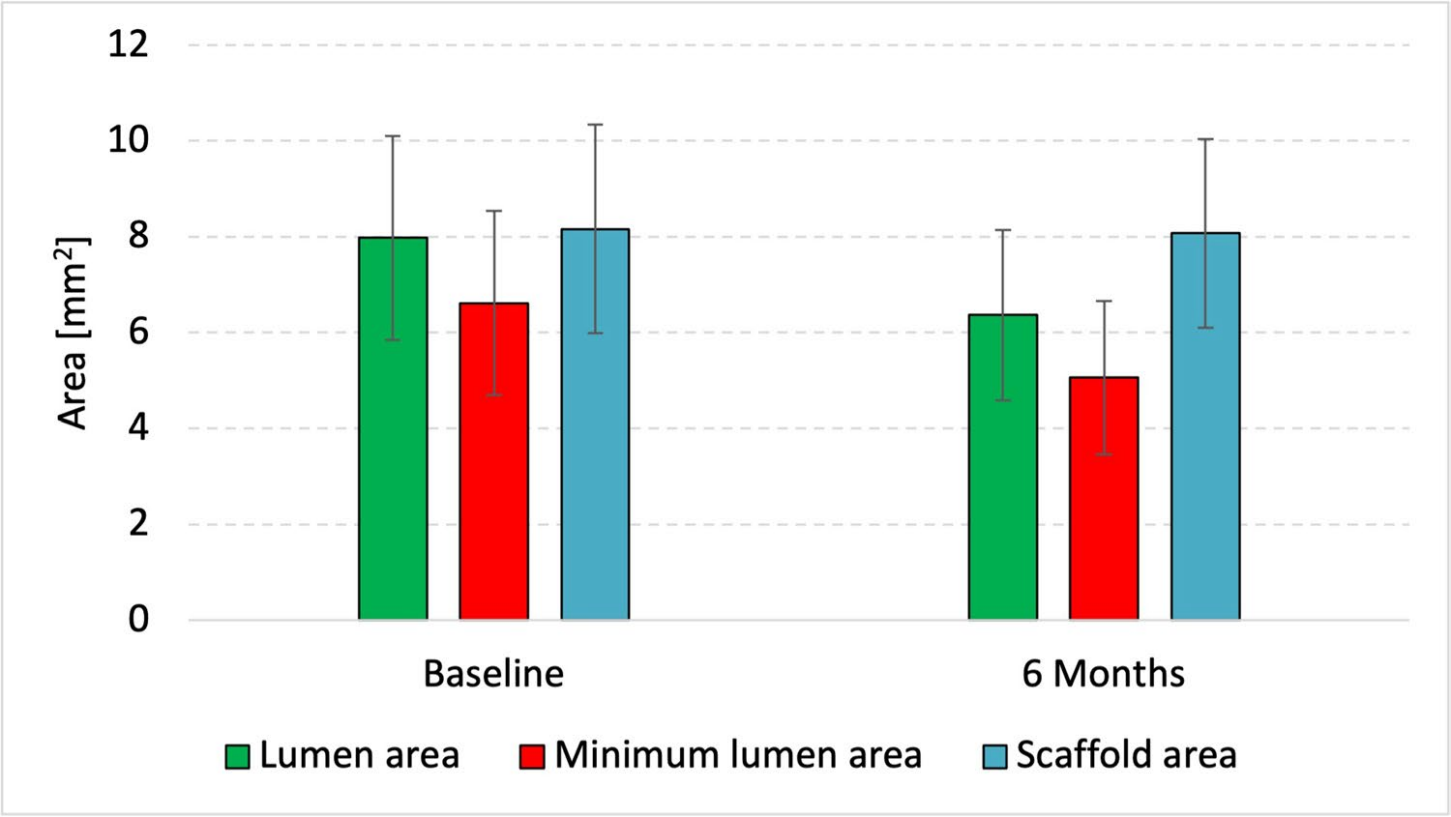
Changes in mean and minimum lumen and scaffold areas between baseline and 6-month follow-up, presented as mean ± standard deviation

The results from quantitative and qualitative OCT analysis at baseline and 180 days are shown in Table 5. Eighteen patients underwent OCT imaging of the index vessel at 6 months. The paired quantitative OCT analysis revealed that the minimum lumen area and the mean lumen area had decreased over the 6 months by 1.55 mm^2^ (6.61 ± 1.92 mm^2^ vs 5.06 ± 1.60 mm^2^, *p* < 0.0001) and by 1.61 mm^2^ (7.97 ± 2.13 mm^2^ vs 6.36 ± 1.78 mm^2^, *p* < 0.0001), respectively. We found no change in the mean scaffold area (8.16 ± 2.18 mm^2^ vs 8.07 ± 1.97 mm^2^, *p* = 0.44) (Fig. 2).

**Table 5:** Quantitative and qualitative optical coherence tomography analysis (paired scaffold level analysis, *n* = 19

	Baseline (post-procedure)	6-month	*p*-value
Quantitative analysis			
Length of scaffold [mm]	22.53 ± 4.21	22.42 ± 4.57	
In-scaffold area			
Minimum lumen area [mm^2^]	6.61 ± 1.92	5.06 ± 1.60	< 0.0001*
Minimum flow area [mm^2^]	6.11 ± 1.61	5.06 ± 1.60	0.0002*
Mean lumen area [mm^2^]	7.97 ± 2.13	6.36 ± 1.78	< 0.0001*
Mean flow area [mm^2^]	7.51 ± 1.95	6.35 ± 1.78	0.0014*
Minimum scaffold area, abluminal [mm^2^]	7.02 ± 2.09	6.83 ± 1.93	0.3146
Mean scaffold area [mm^2^]	8.16 ± 2.18	8.07 ± 1.97	0.4435
Scaffold area obstruction [%]	-	16.25 (12.65–22.47)	-
Deployment index	0.71 (0.66–0.78)	0.74 (0.66–0.79)	1
Expansion index	0.79 (± 0.13)	0.86 (± 0.13)	0.0384*
Periscaffold area			
Distal reference mean lumen area [mm^2^]	6.78 (± 2.77)	6.02 (± 2.30)	0.0340*
Proximal reference mean lumen area [mm^2^]	8.62 (± 3.97)	7.62 (± 2.75)	0.0770
In-scaffold diameter			
Scaffold-to-vessel diameter ratio	1.01 ± 0.01	1.14 ± 0.06	< 0.0001*
Scaffold undersizing	2 (11.76%)	2 (12.50%)^±^	1.00^±^
Scaffold oversizing	0 (0%)	0 (0%)^±^	-
Eccentricity index			
Lumen	0.86 (± 0.02)	0.85 (± 0.03)	0.0919
Scaffold	0.89 (± 0.03)	0.89 (± 0.03)	0.9570
Asymmetry index			
Lumen	0.33 (± 0.07)	0.36 (± 0.07)	0.0195*
Scaffold	0.27 (± 0.06)	0.28 (± 0.06)	0.6070
Neointima			
Mean neointima thickness (μm)	-	177 (161–215)	-
Maximum neointimal thickness (μm)	-	420 (400–590)	-
Neointimal area, on-top of/in-between struts (mm^2^)	-	1.20 (1.01–1.53)	-
Symmetry of the neointima thickness	-	0.23 (0.19–0.29)	-
Malapposition			
Scaffolds with incomplete stent apposition	10 (58.82%)	2 (11.76%)	0.0047*
Malapposed distance, mean [mm]	0.12 (± 0.04)	0.29 (± 0.01)	0.5000
Malapposed area, mean [mm^2^]	0.33 (0.15–0.60)	0.69 (0.30–1.09)	0.5000
Malapposed struts [%]	0.46 (0.00–8.15)	0 (0–0)	0.0020*
Malapposed strut ratio in patients with any malapposed strut [%]	6.30 (1.30–10.91)	1.21 (0.49–1.92)	0.5000
Uncovered struts			
Scaffolds with uncovered struts	-	2 (11.76%)	-
Uncovered struts ratio [%]	-	0 (0–0)	-
Uncovered strut ratio in patients with any uncovered struts [%]	-	5.97 (4.90–7.03)	-
Strut discontinuities, total	3 (17.65%)	2 (11.76%)	0.5637
Uncovered and malapposed	-	0 (0%)	-
Covered and malapposed	-	0 (0%)	-
Uncovered and apposed	-	0 (0%)	-
Covered and apposed	-	2 (11.76%)	-
Healing score	-	0 (0–0)	-
Scaffolds < 2.5 mm in diameter	-	-	-
Scaffolds 2.5–3.5 mm in diameter	-	0 (0–0)	-
Scaffolds > 3.5 mm in diameter	-	-	-
Intraluminal scaffold dismantling	-	0 (0%)	-
Qualitative analysis			
Neoatherosclerosis	-	0 (0%)	-
Calcification	-	0 (0%)	-
Neovascularization	-	0 (0%)	-
Macrophage	-	0 (0%)	-
Lipid	-	0 (0%)	-
TCFA	-	0 (0%)	-
Plaque	-	0 (0%)	-
Thrombus	-	0 (0%)	-
Peri-strut low intensity area (PLIA) analysis			
PLIA score	-	2.00 (0.60–2.71)	-
PLIA arc, per scaffold [°]	-	7.60 (1.38–39.18)	-
PLIA arc			
< 90°	-	0 (0%)	-
90°–180°	-	5 (38.46%)	-
180°–270°	-	2 (15.38%)	-
> 270°	-	6 (46.15%)	-
PLIA intensity	-	13 (76.47%)	
Macrophage arc, per scaffold [°]	-	-	-

Data are presented as mean ± standard deviation, number (%) or median [first quartile, third quartile] unless otherwise indicated

*TCFA*, thin-cap fibroatheroma; *PLIA*, peri-strut low intensity area

^*^p-value < 0.05

^±^*n* = 18

**Fig. 3:**
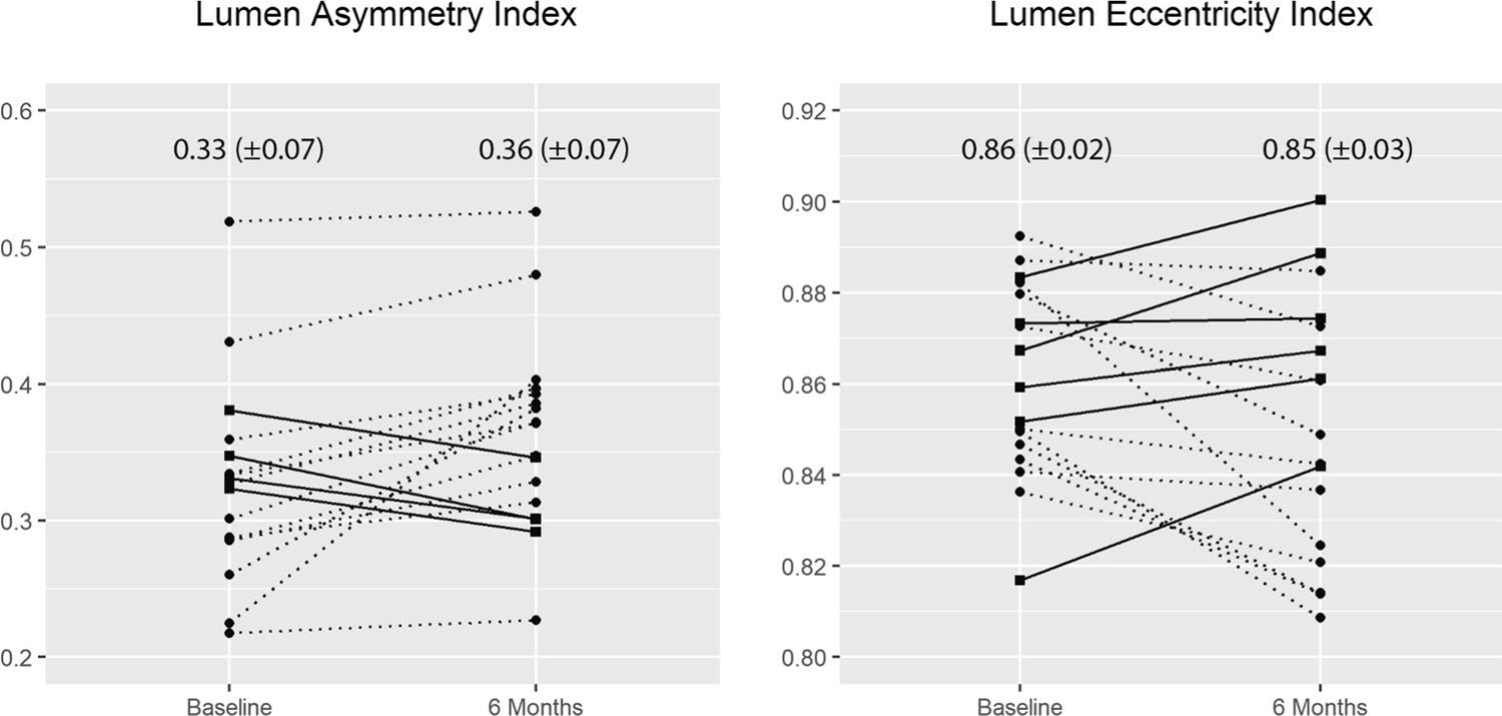
Changes in mean lumen eccentricity index and mean asymmetry index between baseline and 6 months, presented per patient

The distal reference mean lumen area decreased by 0.76 mm^2^ (6.78 ± 2.77 mm^2^ vs 6.02 ± 2.30 mm^2^, *p* < 0.05). There was no statistically significant change in proximal reference mean lumen area (8.62 ± 3.97 mm^2^ vs 7.62 ± 2.75 mm^2^, *p* = 0.08). The number of scaffolds with incomplete stent apposition decreased from 10 (58.2%) to 2 (11.76%) (*p* < 0.05). The rate of malapposed struts decreased from 0.46% at baseline to 0% (*p* < 0.05) 6 months after the procedure. The mean neointima thickness at 6 months was 177 µm (161–215 µm) with 0.23 (0.19–0.29) symmetry. The lumen asymmetry index (AI) decreased by 0.03 (0.33 ± 0.07 vs 0.36 ± 0.07, *p* < 0.05). We observed no significant change in the lumen eccentricity index (EI) (0.86 ± 0.02 vs 0.85 ± 0.03, *p* = 0.09) (Fig. 3).

**Fig. 4:**
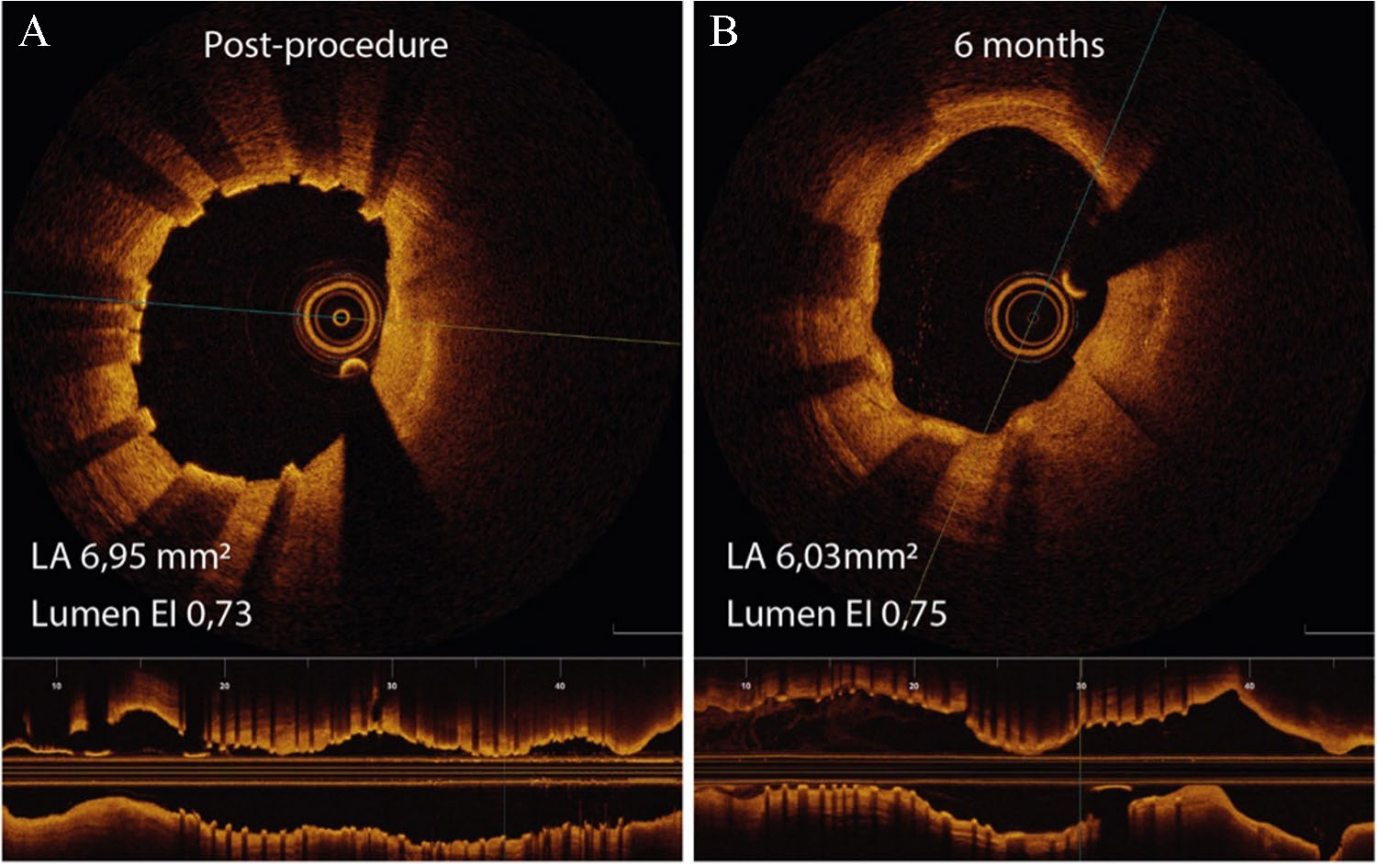
Optical coherent tomography showing a post-procedure intravascular image (**A**) and after an image from 6 months after implantation (**B**). LA, lumen area

None of the scaffolds had any atherosclerotic findings at the 6-month follow-up. The mean peri-strut low-intensity area (PLIA) score 6 months after the procedure was 2.00 (0.60–2.71). An example of the vascular response in OCT is presented in Fig. 4.

## Discussion

The Fantom is a novel third-generation BRS characterised by thin struts, good expansion capability and radiopacity. Most of the studies on the Fantom BRS included patients with stable coronary artery disease. The data on the use of BRS in patients with STEMI are limited, despite the fact that this group of patients may obtain many benefits from BRS implantation. Coronary artery lesions in STEMI patients are often localised in the proximal part of the coronary artery, so restoration of vasomotion may have a greater effect than in patients with stable disease. Due to the necrotic core of the plaque in STEMI lesions, metallic stent implantation interferes with vascular healing and leads to coronary evagination and malapposition. BRS implantation may prevent these complications [15, 16].

The major findings of the current study are as follows:

1. Fantom BRS implantation in patients with STEMI was safe, with no MACEs or DOCEs 6 months after the procedure.
2. We observed an expected steady decrease in minimum lumen area and mean lumen area.
3. OCT showed a decrease in the rate of strut malapposition and an improvement in lumen asymmetry.

Increased platelet activation and vasoconstriction are major concerns resulting from the implantation of a BRS. The results of the Fantom pilot study on 10 patients with STEMI showed that no patient had any DOCEs during a 30-day follow-up period [12]. In the current study, entirely guided by OCT, we observed that Fantom BRS was safe. The primary DOCE—consisting of cardiac death, target vessel-related MI and TLR—did not occur during the 6 months of the study. The TROFI II study compared the safety and efficacy of the first-generation Absorb stents and the metallic everolimus-eluting stent in patients with STEMI. Within 6 months of implantation, DOCE occurred in 1 patient (1.1%) in the Absorb arm; it was a subacute definite stent thrombosis leading to MI and clinically-driven TLR [17]. Also, there were no MACEs in our study and no cases of stent thrombosis. In the Fantom II study with 108 patients, 3 MACEs (2.8%) were recorded with 1 case (0.9%) of stent thrombosis [18].

Despite MLA and mean lumen area decreasing after 6 months, the Fantom BRS maintained a stable minimum and mean scaffold area. Similar results were obtained in the FANTOM II study [19].

According to Asano et al., LLL may be a predictor of TLR in patients after various angioplasty techniques. The Kaplan–Meier analysis showed that the incidence rate of TLR in the patients with an LLL over 0.50 mm was 24.6% at the 4-year follow-up angiography, while in the patients with an LLL of 0.50 mm or greater was 4.6% [20]. In our study, the LLL was 0.20 mm ± 0.25 mm, while in the Fantom II study, it was 0.25 ± 0.40 mm [18]. The LLL values at 6 months with metallic DESs were 0.11 ± 0.23 mm with everolimus-eluting stents [21], 0.36 ± 0.48 mm with paclitaxel-eluting stents [22] and 0.61 ± 0.46 mm with zotarolimus-eluting stents (after 8 months) [23].

In the present study, the frequency of PLIA was 76.47% with a mean value of 2.00 (0.60–2.71). This may be related to arterial healing and local vessel injury. Sato et al. suggested that the appearance of PLIA on OCT up to 1 year after BRS implantation may be related to the biological processes occurring within the scaffold, as with DESs. This may resolve after 3 or 4 years, when the resorption process has been completed, as opposed to metallic stents [24].

There are obvious limitations due to the single-arm, non-randomised study design and the limited number of patients in one centre. However, to date, this is the only longitudinal study with serial, long-term OCT follow-up of the Fantom BRS in STEMI patients.

In conclusion, serial 6-month OCT imaging after implantation of the third-generation Tyrocore-based bioresorbable coronary scaffold showed no significant safety concerns, with good coverage of the struts along with excellent healing of the scaffold and low neointima growth and no signs of neoatherosclerosis. Still, given a small study sample, these results warrant to be confirmed in a larger trial, prior drawing firm conclusions.

## Impact on Daily Practice

Implantation of Fantom BRS is related to a low risk of occurrence of MACE or DOCE and scaffold thrombosis. The resorption of the scaffold with a low neointima growth may improve long-term outcomes and future treatment for patients.

## Data Availability

The datasets generated during and/or analysed during the current study are available from the corresponding author on reasonable request.
